# Parallelization and optimization of genetic analyses in isolation by distance web service

**DOI:** 10.1186/1471-2156-10-28

**Published:** 2009-06-19

**Authors:** Julia L Turner, Scott T Kelley, James S Otto, Faramarz Valafar, Andrew J Bohonak

**Affiliations:** 1 Computational Science Research Center, San Diego State University, San Diego, California 92182, USA; 2 Department of Biology, San Diego State University, San Diego, California 92182, USA; 3 Bioinformatics and Medical Informatics Graduate Program, San Diego State University, San Diego, California 92182, USA

## Abstract

**Background:**

The Isolation by Distance Web Service (IBDWS) is a user-friendly web interface for analyzing patterns of isolation by distance in population genetic data. IBDWS enables researchers to perform a variety of statistical tests such as Mantel tests and reduced major axis regression (RMA), and returns vector based graphs. The more than 60 citations since 2005 confirm the popularity and utility of this website. Despite its usefulness, the data sets with over 65 populations can take hours or days to complete due to the computational intensity of the statistical tests. This is especially troublesome for web-based software analysis, since users tend to expect real-time results on the order of seconds, or at most, minutes. Moreover, as genetic data continue to increase and diversify, so does the demand for more processing power. In order to increase the speed and efficiency of IBDWS, we first determined which aspects of the code were most time consuming and whether they might be amenable to improvements by parallelization or algorithmic optimization.

**Results:**

Runtime tests uncovered two areas of IBDWS that consumed significant amounts of time: randomizations within the Mantel test and the RMA calculations. We found that these sections of code could be restructured and parallelized to improve efficiency. The code was first optimized by combining two similar randomization routines, implementing a Fisher-Yates shuffling algorithm, and then parallelizing those routines. Tests of the parallelization and Fisher-Yates algorithmic improvements were performed on a variety of data sets ranging from 10 to 150 populations. All tested algorithms showed runtime reductions and a very close fit to the predicted speedups based on time-complexity calculations. In the case of 150 populations with 10,000 randomizations, data were analyzed 23 times faster.

**Conclusion:**

Since the implementation of the new algorithms in late 2007, datasets have continued to increase substantially in size and many exceed the largest population sizes we used in our test sets. The fact that the website has continued to work well in "real-world" tests, and receives a considerable number of new citations provides the strongest testimony to the effectiveness of our improvements. However, we soon expect the need to upgrade the number of nodes in our cluster significantly as dataset sizes continue to expand. The parallel implementation can be found at .

## Background

According to the National Institutes of Health, by 2005 the rate of DNA sequence submission to National Center for Biotechnology Information's (NCBI) GenBank database increased to approximately 3 million new sequences each month or 4,000 sequences per hour [1], and the rate of deposition has continued to accelerate ever since. As more genetic information becomes available, the demand for more computing power to analyze this information grows proportionally. Although CPU speed continues to increase at a remarkable rate, the large quantity of sequence data has outpaced the rate at which computer hardware is improving. The idea, popularized by Gordon Moore [2], that the processing speed of sequential computers doubles every two years is insufficient to keep up with the expanding complexity of genetic information. Thus, optimization and parallel processing need to be employed in order to develop algorithms that offer significantly more efficient data processing.

Parallelization coupled with optimization has been particularly effective in speeding up the most heavily used bioinformatics tool NCBI BLAST [3]. BLAST has a web interface that makes it accessible to the widest possible array of users. Web interfaced tools are popular among biologists because they are easy to use, require only a web browser to execute, and typically return valuable information in an intuitive format. BLAST has been adapted to handle the influx of data with efficient search algorithms and several approaches for parallel processing [4,5]. Asterias, ParaMEME, and CBSU's Web Computing Interface [6-8] are also web-based bioinformatics analysis tools that have improved processing time by subdividing work among multiple processors. For example, CSBU has tools specifically of interest for population and evolutionary genetics analysis (e.g., MrBayes, Parentage, PLINK).

Like CSBU, the Isolation by Distance Web Service (IBDWS) is a web-based program that performs statistical analysis with a user-friendly interface for population genetics [9]. Statistical analysis can be performed on the relationships among individuals, or by grouping sets of individuals into populations a priori. IBDWS is generally designed to perform statistical tests on the latter. The website is named after "Isolation by distance" (IBD), a population genetics principle first described by Sewall Wright [10]. IBD describes patterns in allelic frequencies that are the consequence of spatially restricted gene flow, specifically an increase in the genetic distance between pairs of populations as the geographic distance between them increases. Two separate methods (the Mantel test and Reduced Major Axis (RMA) regression) are used to determine the correlation between genetic distance and geographic distance. The Mantel algorithm tests for non-random associations between a genetic distance matrix and a matrix containing geographic distances [11]. As described by Bohonak [12], the RMA regression quantifies the strength of the IBD relationship, with slope and intercept errors calculated through a variety of resampling techniques.

IBDWS arose as a conversion of the standalone Isolation by Distance program for Macintosh and Windows [9,12]. IBDWS has progressively become more flexible through its later versions (e.g., the ability to directly input raw DNA data sets in v. 3.0). The conversion to IBDWS in 2004 allowed many users to process more data faster than before and there was no other easily accessible web-based software available for exploring IBD patterns. The papers published in 2004 that cited IBDWS had an average population size of 16, with the largest 10% having an average of 34 populations [13-23]. Between November 2006 and November 2007 we tracked the number of populations in the data sets used in IBDWS. Out of 7947 analyses, the average number of populations was 23, with the largest 10% having an average of 95 populations. As the number of populations increases, the complexity of the calculations increases and the time to process submitted data sets grows exponentially.

In this paper, we set out to improve the speed and performance of IBDWS using a combination of algorithmic optimization techniques and parallel computing. Even after algorithmic optimizations, the growing size of submitted data sets is already too large for rapid analysis on a single server. For example, in the last implementation prior to these modifications, 100 populations with 10,000 randomizations required at least 7 hours to complete. In order to improve analysis speed, we first identified the time intensive parts of the program and determined their time complexity. Randomization routines within the Mantel test and RMA calculations proved particularly time-consuming but amenable to algorithmic optimization and parallelization. Accordingly, we algorithmically combined the Mantel test and RMA randomizations, distributed the randomizations among several processors, and implemented the Fisher-Yates shuffling algorithm [24] for conducting the randomizations. The differences in runtime between each algorithm enhancement, the overall runtime changes, the predicted speedup, and the effects of increasing the number of nodes are discussed.

## Implementation

**Figure 1:**
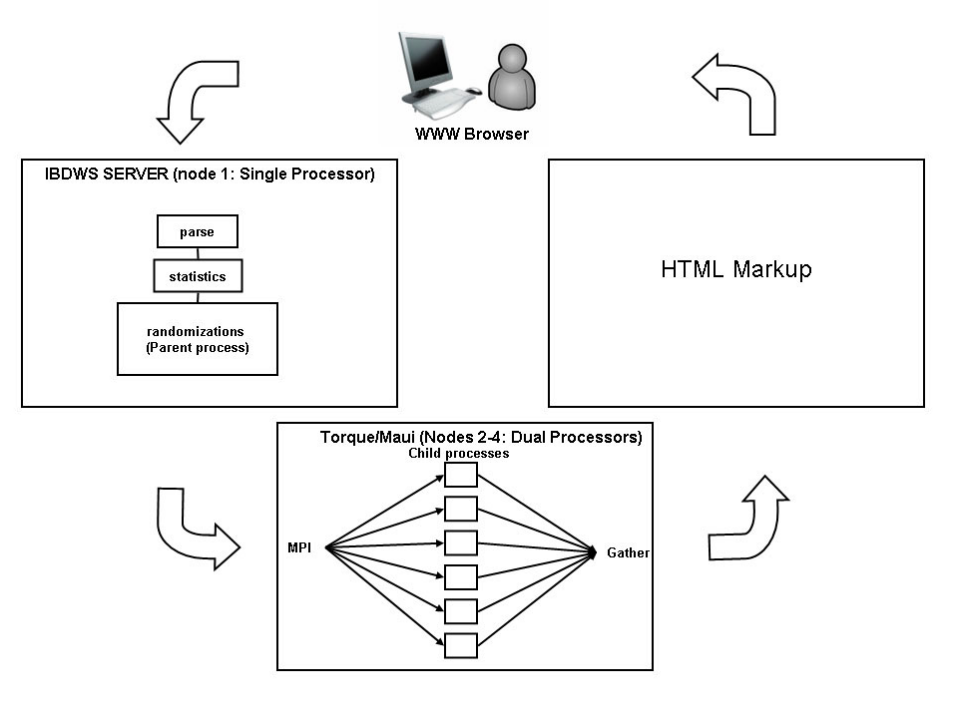
**Parallel IBDWS**. Diagram of parallel IBDWS showing input, parallelization and data return.

The parallel implementation of IBDWS is a straight-forward master slave configuration diagrammed in Figure 1. The parent node completes initial calculations and then sends the resulting values to each child node. Each child independently runs a section of code and then returns the results to the parent node. The flow of information in parallel IBDWS is as follows: (1) the user inputs data, (2) the data are parsed, (3) statistical calculations are performed, (4) the parent node initializes randomizations to generate null distributions for the statistical calculations, (5) the randomizations are equally divided among child nodes, (6) the randomization results are returned to the parent, (7) the parent sorts the randomizations results and calculates statistical significance, and (8) a HTML page displays the text-based and graphical results for the user.

### Runtime Analysis

**Figure 2:**
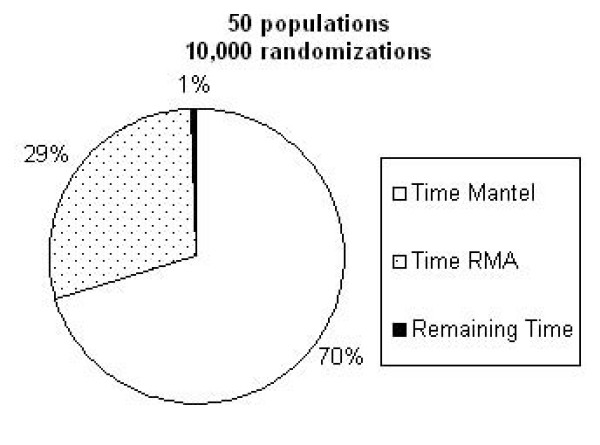
**Runtime analysis**. Runtime analysis of the original version of IBDWS, demonstrating that the majority of the processor time was spent performing the RMA and Mantel tests.

In order to implement the most effective parallelization strategy, we first determined what aspects of the analysis required the most CPU time. Runtime tests revealed that approximately 99% of the analysis time was consumed by the sections of code that perform the RMA regression and the Mantel tests (Figure 2). The remaining 1% is involved in tasks such as parsing input, calculating genetic distances from raw data, and generating output. None of these would benefit from parallel processing.

IBDWS is designed to analyze the relationship between a lower triangular matrix of genetic distances between all pairs of populations, and the corresponding geographic distance matrix. The statistical significance of this relationship is tested using a Mantel test [11], where the test statistic Z is calculated as:



A refers to the matrix of genetic distances, B is the matrix of geographic distances, and N is the dimensionality of two matrices. The correlation coefficient r is obtained by:



with sA and sB representing the standard deviation of A and B respectively. The r value can range from -1 to 1. The rows of matrix A can then be randomly permutated to create a null distribution to calculate the probability that the observed r and Z occur under a null hypothesis of no relationship. Since each row and column (corresponding to a single population) is treated as a functionally linked group during the randomizations, the Mantel test is more suitable than other statistical tests which operate under the assumption that each population pair is independent. The major time consumption in this section of code is the shuffling to randomize matrix A O(n^2^), and the loop to calculate Z and r in each iteration O(n).

The RMA regression is the second most computationally intensive section of the code. RMA regression is used to estimate the linear slope and intercept of the IBD relationship using standard regression approximations:





The coefficient of determination, *r*^2 ^is found by:



Bootstrapping and jackknifing re-sampling methods are both used to measure error in the RMA regression, since groups of points corresponding to contrasts with the same population are not independent in matrix-based analyses such as these. The bootstrap resampling requires the most time in this section of the code, with O(n) time complexity. Both the RMA and Mantel test can be run up to four times, if various logarithmic data transformation options are chosen.

### Speedup Strategy

Reducing the processing time for IBDWS involved three steps: (1) combining the loop that bootstraps for RMA and the overall randomization loop for the Mantel test, (2) changing the algorithm that randomly shuffles matrix A in the Mantel test, and (3) splitting the combined RMA Mantel randomization loop among multiple processors.

### Serial Optimization

The Mantel and RMA algorithms performed similar randomization loops in separate locations of the code, with the number of randomizations chosen by the user. Combining the loops decreased loop overhead and therefore improved runtime. However, since loop overhead is only a constant multiplicand, time complexity remains at O(n) after this improvement. This optimized version of the randomizations will be referred to as the combined randomization.

The original code utilized an inefficient "unoptimized" shuffling algorithm that shuffled the rows and columns of matrix A by randomly selecting a number between 1 and N, placing it in a new array, and then checking to see whether that number had previously been used in the new array. This algorithm took n times to go forward and n times to check backward for a complexity of O(n2), much like a modified bubble sort. (In bubble sort, each element is compared to insure in sequential order, and in this case each element is compared to insure random order.) We implemented the Fisher-Yates shuffling algorithm to reduce the time spent shuffling the matrix down to O(n). With the Fisher-Yates method, each element is swapped with a random element in the matrix [24]. This reduced the time complexity because it was no longer necessary to check previous elements as in the unoptimized shuffling algorithm.

### Parallel Optimization

Further improvements were also made by exploiting parallelism in the algorithm. The architecture of parallel IBDWS consists of seven total processing elements. The parent node submits data sets with a large number of populations (defined later) to the batch processor, which queues jobs to run on the other 6 other processors. Therefore, small jobs never overlap larger jobs. Small jobs only run on the parent node and do not need to wait for larger jobs to finish processing.

The combined randomization proved ideal for parallelization, as it contained independent iterations with no loop-carried dependences. To implement multi-node processing, the randomizations were divided equally to each Processing Element (PE). If the number of randomizations did not divide among the number of PEs, an additional randomization was added to some PEs to achieve equality, although these were ignored in later calculations. If the user chooses fewer randomizations than the number of PEs (which is unlikely), then each PE performs one randomization and the additional randomizations are discarded in later calculations. Each PE was tasked with calculating bootstrap pseudoreplicates for both the Mantel test and RMA regression. MPI was initialized at the point of bootstrapping. When the bootstrap iterations completed MPI_Gather was used to combine the arrays from each PE into one large array for further calculations.

### Experimental Design

To evaluate the processing enhancements of parallel IBDWS, we compared runtimes to the serial version of IBDWS. The algorithmic improvements were also tested for the single processor version. The results of our tests allowed us to set a cutoff for the number of populations required for parallel processing, calculate speedup, and examine scalability.

To determine if parallel IBDWS was an improvement over the previous serial version, two variables were considered when creating the data sets: the population size and the number of randomizations performed. Five data sets of size 10, 20, 30, 40, and 50 populations were randomly generated by creating one large matrix of values. Because all data sets were derived from this single matrix, the smallest data set (a 10 population matrix) was a subset of each of the larger data sets, reducing possible bias. Each data was analyzed using 100, 1000, and 10000 randomizations. To evaluate the behavior of the system for extremely time intensive data, we additionally ran two very large data sets of 100 populations and 150 populations with 10000 randomizations. These final two tests represent the size of data sets that we predict will become typical for IBDWS within the next few years.

The data used in these tests were input as user-provided genetic distances and geographic distances. IBDWS also accepts raw data in the form of diploid genotypes or DNA sequences. We did not analyze these raw data set types, because they take relatively little additional time, and are irrelevant to optimization and parallelization of the code.

### Implementation and Platform

IBDWS was written predominately in C++ with MPI code running on Apache 2.2.0. The C++ code was compiled with a g++ and mpich-1.2.7 compilers. In addition to C++, scripting language Perl 5.8.8 was used to initialize the batch processor. Webpage data were parsed via cgic (a publicly available C++ code) [25]. Images were created with CGraph (a C++ plotting library). Distributed processing resources were managed by TORQUE 2.1.6, a predecessor of OpenPBS, using Maui 3.7.6p17 scheduler [26]. We have implemented IBDWS on a cluster of 4 nodes. The parallel compute nodes were three dual core AMD Opteron Processor 246 with 2.0 GHz and 3 GB of RAM. The fourth node was an AMD Athlon 64 Processor 3500+ with 2.2 GHz and 3 GB RAM, which was the web server and processed the small serial jobs. The code can be downloaded at sourceforge.net: 

## Results and Discussion

### Algorithmic improvements as a function of time

**Figure 3:**
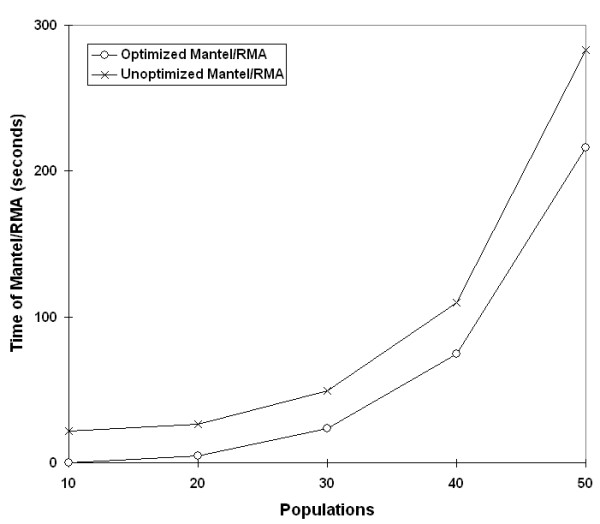
**Effect of loop randomization combination by population size**. Speed improvements from combining the RMA and Mantel randomizations into a single loop. Data sets of various population sizes were analyzed with 10,000 randomizations.

**Figure 4:**
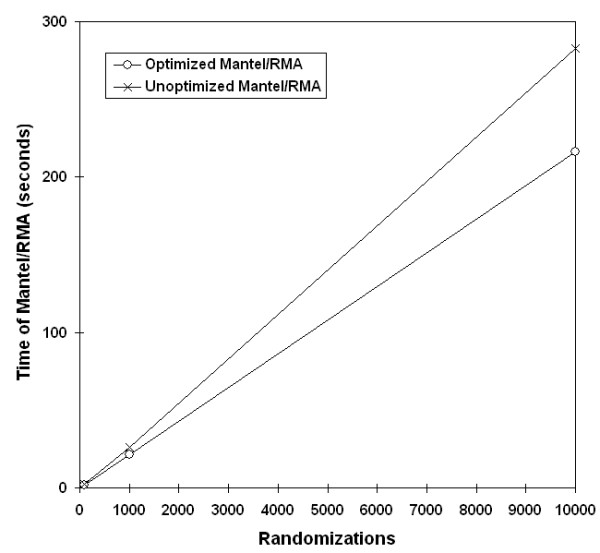
**Effect of loop randomization combination by number of randomizations**. Tests of combined loop randomizations with a data set of 50 populations, analyzed with 100, 1000, and 10,000 randomizations.

Initial experiments were performed to test the two code enhancements prior to parallelization: Mantel/RMA combined and Fisher-Yates shuffling. First, the method of combining the RMA and Mantel randomization loops into one loop was tested with the expectation that the amount of time should decrease, but overall time complexity should stay the same at O(n). (For details on how we calculated the expected speedup improvements, see Additional File 1 – Time complexity analysis.) The runs behaved as expected for a range of population sizes and number of randomizations (Figure 3, 4). As the number of populations increased, the time was consistently less for the combined loop at every point (Figure 3), and also lower for 1000 and 10,000 randomizations (Figure 4).

**Figure 5:**
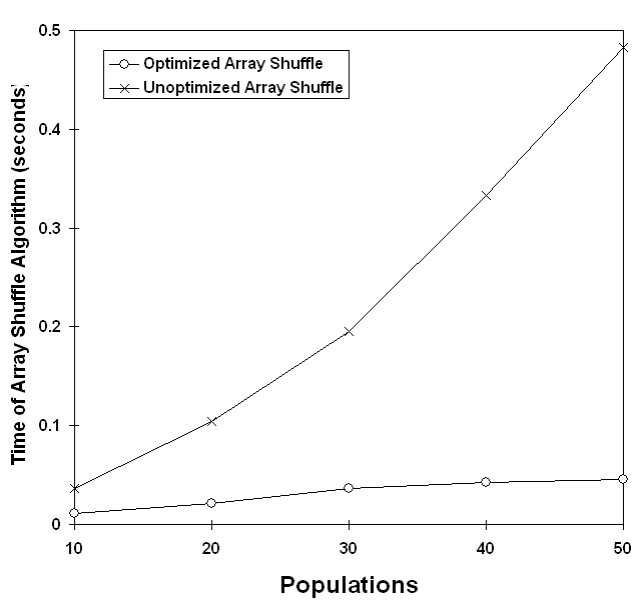
**Effect of Fisher-Yates shuffling by population size**. Changes in time complexity from exponential to linear after implementation of Fisher-Yates shuffling. Data sets of various population sizes were analyzed with 10,000 randomizations.

**Figure 6:**
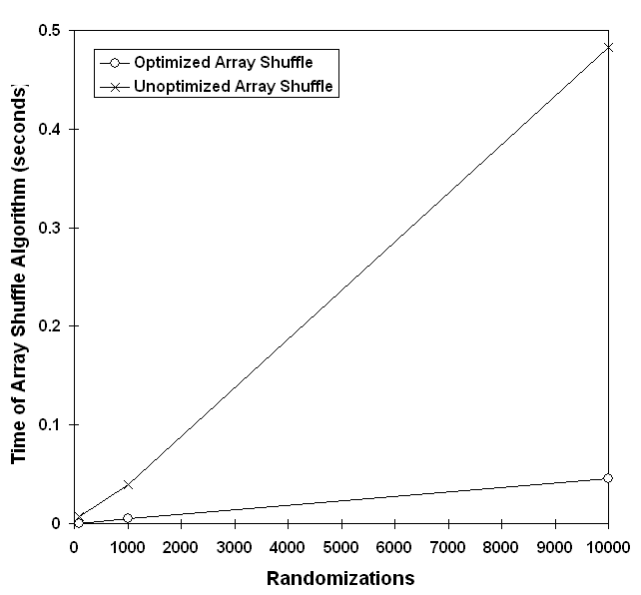
**Effect of Fisher-Yates shuffling by number of randomizations**. Implementation of Fisher-Yates shuffling was tested with a data set that included 50 populations analyzed with 100, 1000, and 10,000 randomizations.

Next, run times were compared before and after implementation of the Fisher-Yates matrix shuffling in the Mantel test. Unlike the previous code optimization, we predicted that the unoptimized shuffling algorithm would have exponential growth with increasing populations, and the optimized Fisher-Yates algorithm would be linear. The timing of data sets demonstrated trends as expected (Figure 5, 6).

### Comparing parallel configuration

**Figure 7:**
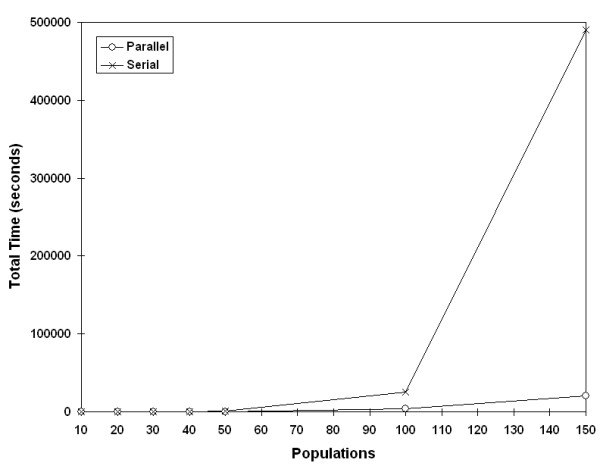
**Effect of parallelization by population size**. Time complexity improvement of parallelization with test population sizes greater than 30 (10,000 randomizations).

**Figure 8:**
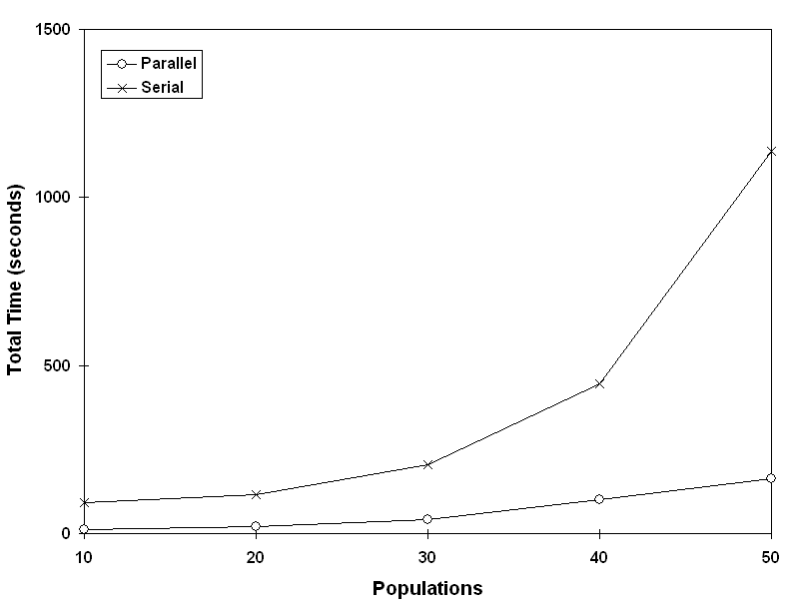
**Effect of parallelization at small population sizes**. Effects of parallelization strategy with small population sizes (10,000 randomizations).

One of the goals of timing the parallel and serial code was to determine at what point the analyses benefited from parallelization. Timing tests revealed that data sets with greater than 30 populations and more than 100 randomizations would benefit from parallelization, given the CPU speed (data not shown). Once a cutoff was set, the final configuration was determined with seven PEs configured as previously described. We compared this setup to the serial program and found that the time savings becomes more dramatic with larger population sizes (Figure 7, 8). (With regards to the user interface, the switch between one processor and multiple processors is completely transparent.)

### Speedup

**Figure 9:**
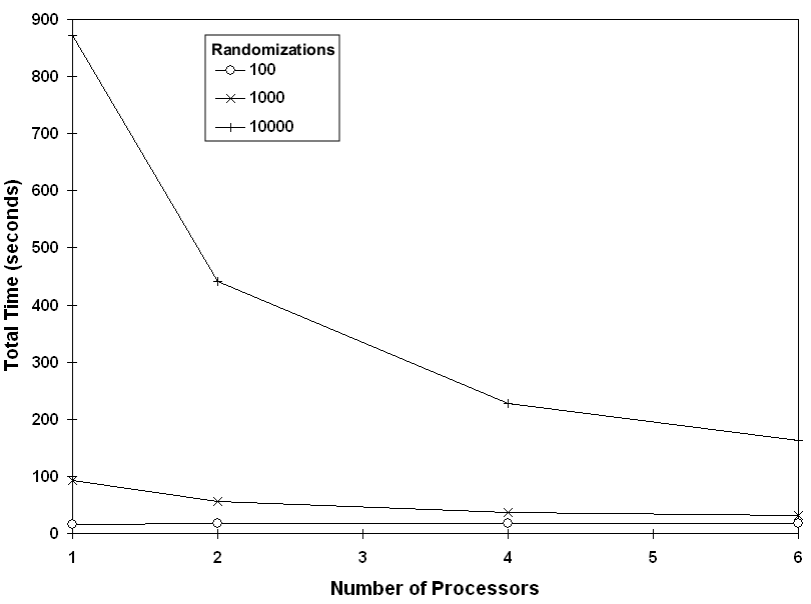
**Effect of adding processors by number of randomizations**. Reduction in runtime with the addition of processors reduces, for a data set with 50 populations.

**Figure 10:**
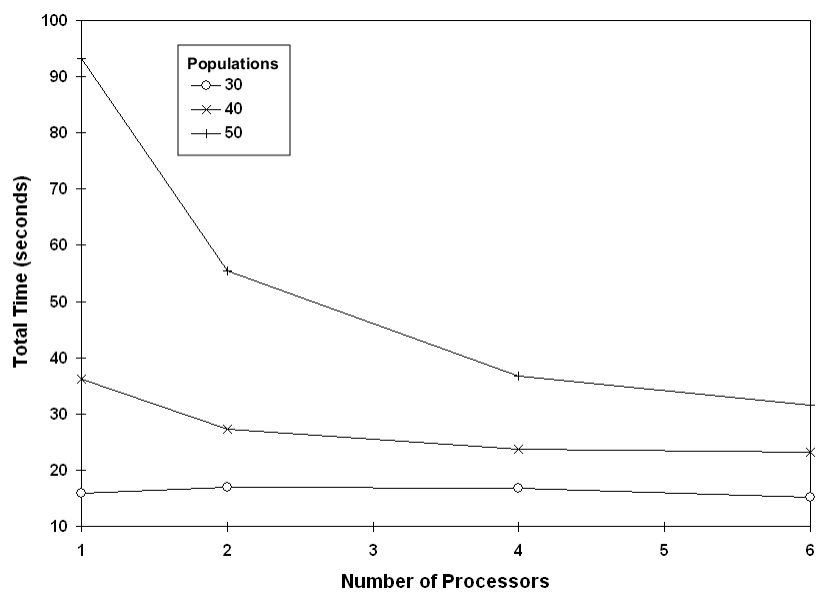
**Effect of adding processors by population size**. Effect of adding processors on runtime with populations of differing sizes (1000 randomizations).

In addition to comparing the complete cluster against a single processor, we compared the performance of our code against increasing numbers of processors. Again, time decreased for across the range of data set sizes and number of randomizations (Figure 9). For each processor added to parallelization we saw an improvement, although resources limited us to only testing a maximum of six processors (Figure 10).

**Figure 11:**
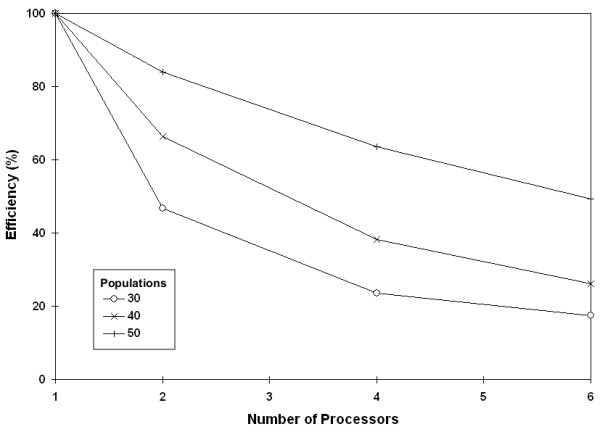
**Efficiency analysis**. Efficiency percentage as a function of increasing population size. Efficiency increased, approaching linearity, as the number of populations increased.

Ideally, the speedup of serial time over parallel time should be linear. In our experiments, we found that the speedup becomes more linear as the number of populations increases (data not shown). Relationships for data sets with the most populations are more linear, because the efficiency decreases more dramatically for smaller populations (Figure 11). However, the efficiency of 50 populations remained above 80% for all processors divisions, which is promising for future needs to analyze greater numbers of populations with more processors. Careful future testing will be needed to determine the optimal number of processors for data sets of various sizes.

To assure that no computational errors were inadvertently introduced when modifying the source code, we ran 84 test data sets via a web browser from an external computer. These runs constituted 3–6 replicates from each cell of a fully factorial design with 2 IBDWS versions (serial or parallel), 2 data sets (10 or 100 populations), 3 levels of randomization (100, 1000 or 10000 randomizations), and containing/lacking an indicator matrix. (The indicator matrix, which is an optional component of submitted data sets, is essentially a second independent variable. Presence of the indicator variable triggers an additional set of Partial Mantel Test analyses.) We verified that seven parameters did not change between serial and parallel versions of the program. These parameters were four point estimates (Mantel Test r, Partial Mantel Test r, RMA intercept, RMA slope) and four output parameters generated from randomizations (p-values for Mantel Test and Partial Mantel Test, and lower/upper bounds from 95% CI of RMA intercept using the "bootstrap over all points" option). For any particular input data set, the four randomly generated output parameters did not vary significantly (p-values varied from 0.11 to 0.97 across eight ANOVAs), and none of the four point estimates changed in any run.

## Conclusion

We have developed a parallel method for data analysis in the Isolation by Distance Web Service program (IBDWS). A logical approach to code optimization and parallel processing of the data yielded notable improvements in response time, particularly for large data sets. Our algorithmic changes improved the time complexity of the shuffling algorithm from O(n^2^) to O(n), eliminated redundancy in the code, and allowed for seamless parallelization. On our small cluster we encountered larger improvements. For instance, a seven hour run of 100 populations and 10,000 randomizations on the serial IBDWS was reduced to one hour on the parallel implementation. We found that these improvements are in concordance with our theoretical time complexity calculations. The unoptimized program had run times that increased exponentially as the populations increased, which would be correct with the population being squared in the estimate of O(kp^2^). When the program was algorithmically optimized the shuffle algorithm no longer had exponential growth with increasing populations and the growth was more linear, which matches a time complexity of O(kp). The final theoretical time complexity we calculated of O(pN) for the parallel version, also could be seen in our runtime analysis when incrementally increasing the number of processors. The speed of the program is dependent on the number of processors. The theoretical calculations however fail to take into account the time to transport information, so small datasets can not be divided infinitely without increasing the processing time. We expect that this will increase the usability to researchers, and immediately after updating IBDWS to the parallel version, users were analyzing data sets as large as 200 populations with increased speed. Future directions of this research will include transition to a larger computational cluster and implementing methods to detect non-linear IBD patterns.

## Availability and requirements

**Project name**: Isolation by Distance Web Service

**Project home page**:  and 

**Operating system(s)**: Available using any web browser at the URL above.

**Programming language**: Python, C++

**Other requirements**: No installation requirements. Users can test the website using sample data available at the project homepage.

**License**: GPL Version 3

**Any restrictions to use by non-academics**: None

## Authors' contributions

JLT completed most of the design, implementation, testing and debugging of the novel software and algorithms in this study. JLT also produced all the data, created the figures and wrote the majority of the paper. STK assisted with overall study and algorithm design, manuscript preparation, editing of multiple drafts and submission of the final version of the manuscript. JSO provided key expertise on web administration and helped compile the code on the cluster. FV assisted with all time complexity calculations (Additional File 1), including estimating expected improvements and determining efficiency. AJB wrote the original version of IBD and oversaw debugging, project direction and the editing of the manuscript. All authors have read and approved the final manuscript.

## Supplementary Material

Additional file 1 **Time Complexity Analysis**. A thorough description of the time complexity analysis, including expressions for the unoptimized and optimized serial and parallel code. Click here for file
