# Supplementary material for: Parallelization and optimization of genetic analyses in isolation by distance web service
Source: BMC Genet. 2009 Jun 19;10:28. doi: 10.1186/1471-2156-10-28 (PMC2713274; doi:10.1186/1471-2156-10-28)
Supplement: Additional file 1 — Time Complexity Analysis. A thorough description of the time complexity analysis, including expressions for the unoptimized and optimized serial and parallel code. [file 1471-2156-10-28-S1.doc]

### Additional file 1 – Time Complexity Analysis

The basic outline and time complexity of each section is described below. Variable k, p, and N denote number of randomizations, number of populations, and number of processors. The outline of the unoptimized serial code is a follows:

(1) Initial Calculations

(2) For (i = 0; i<k; i++) {

(3) Shuffle Algorithm

(4) Mantel Test Calculations

}

(5) For (i = 0; i< k; i++) {

(6) RMA Calculations

}

This will result into the following time complexity expression:

The outline of the optimized version of this code is as follows:

(1) Initial Calculations

(2) Test number of populations

(3) For (i = 0; i< k; i++) {

(4) Optimized Shuffle Algorithm

(5) Mantel Test Calculations

RMA Calculations

}

This leads into the following time complexity derivation for this code:

Therefore, theoretical speedup of the optimized serial code verses the unoptimized serial code is O(p):

.

This means that speedup should increase linearly with the number of populations. So, the higher the population number, the more noticeable the speedup of the optimization.

Large populations in Figure 4 use the parallel version of the optimized code which is outlined in the following:

(1) Initial Calculations

(2) Test number of populations

(3) Print data to file

(4) Start MPI multiple node processing

(5) For (i = 0; i< slice = ; i++) {

(6) Optimized Shuffle Algorithm

(7) Mantel Test Calculations

(8) RMA Calculations

}

The time complexity upper bound of the parallel code can be derived by the following:

Therefore, theoretical speedup of the parallel code verses the unoptimized serial code is O(pN):

.

Here speedup is a factor of number of populations and the number of processors available to the parallel code. Theoretical speedup of the parallel code verses the optimized serial code is O(N):

.

The theoretical speedup increases linearly with the number of processors available. This increase is bounded by communication overhead (not included in above calculations) which in the limit will eliminate the benefits if having more processors. Notice from the figures when data are analyzed in parallel, they must be written to files. Files need to be generated because the main code does not share memory with the parallel section. Values are transferred by reading in data from files.
